# Production of Greener High-Strength Concrete Using Russian Quartz Sandstone Mine Waste Aggregates

**DOI:** 10.3390/ma13235575

**Published:** 2020-12-07

**Authors:** Aleksandr Tolstoy, Valery Lesovik, Roman Fediuk, Mugahed Amran, Murali Gunasekaran, Nikolai Vatin, Yuriy Vasilev

**Affiliations:** 1 Department of Building Materials Science, Products and Structures, Belgorod State Technological University named after V.G.Shoukhov, 46 Kostiukova Str., 308012 Belgorod, Russia; tad56@mail.ru (A.T.); naukavs@mail.ru (V.L.); 2 Research Institute of Building Physics, Russian Academy of Architecture and Construction Sciences, 21 Lokomotivny pr., 127238 Moscow, Russia; 3 Polytechnic Institute, Far Eastern Federal University, 690922 Vladivostok, Russia; 4 Department of Civil Engineering, College of Engineering, Prince Sattam Bin Abdulaziz University, Alkharj 11942, Saudi Arabia; 5 Department of Civil Engineering, Faculty of Engineering and IT, Amran University, Amran 9677, Yemen; 6 School of Civil Engineering, SASTRA Deemed to be University, Thanjavur 613404, India; murali@civil.sastra.edu; 7 Higher School of Industrial, Civil and Road Construction, Peter the Great St. Petersburg Polytechnic University, 195251 St. Petersburg, Russia; vatin@mail.ru; 8 Department of Road-Building Materials, Moscow Automobile and Road Construction University, 125319 Moscow, Russia; yu.vasilev@madi.ru

**Keywords:** greener high-strength concrete, quartz sandstone, mine waste, aggregate, compressive strength, freeze–thaw resistance

## Abstract

Quartz sandstone (QS) is a mine waste; therefore, its use in construction allows for both reducing the cost of the concrete and contributing to the utilization of waste. The scientific originality of this study is the identification of models of the effect of QS aggregate on the physicomechanical, durability characteristics, and eco-safety of greener high-strength concrete. The study used an energy-efficient method of non-thermal effects of electromagnetic pulses on the destruction mechanisms of quartz-containing raw materials. The characteristics of quartzite sandstone aggregates, including the natural activity of radionuclides, were comprehensively studied. The features of concrete hardening, including the formation of an interfacial transition zone between the aggregate and the cement matrix, were studied, taking into account the chemical and morphological features of quartzite sandstone. In addition, the microstructural and morphological properties of concrete were determined after a 28 day curing. In this study, the behaviors of the concrete with QS aggregate were investigated, bearing in mind the provisions of geomimetics science on the affinity of structures. The results obtained showed that the QS aggregate had the activity of natural radionuclides 3–4 times lower compared to traditional aggregates. Efficient greener concrete with a 46.3 MPa compressive strength, water permeability grade W14, and freeze–thaw resistance of 300 cycles were also obtained, demonstrating that the performance of this greener concrete was comparable to that of traditional concrete with more expensive granite or gabbro diabase aggregates.

## 1. Introduction

The current stage of the development of civilization is characterized by a deteriorating environmental situation, a lack of energy resources as well as natural and technological disasters [1,2,3]. A person spends a significant part of his time surrounded by building materials that are designed to protect him from the negative effects of the environment [4,5,6]. Natural or artificial aggregates are the main part (up to 90% by volume) of concrete and mortars; therefore, their quality and properties are largely determined by aggregates [7,8]. The problem arises in the difference in properties of aggregates obtained even from the same rock. For example, to save the binder, it is necessary that the strength of the aggregate is 1.2–1.5 times higher than the design class of concrete. This indicator mainly depends on the density and structure of the aggregate [9]. The operational properties of aggregates are determined by the mineral and chemical compositions and water and frost resistance. The important characteristics of the aggregates also include the shape of the grains, the nature of the surface, structure, chemical composition, as well as economic indicators.

Many authors have worked on research to develop innovative sustainable concrete and/or mortar utilizing some aggregates. Longo et al. [10] obtained lightweight solutions based on geopolymers for structural and energy upgrading of buildings. Cobo Ceacero et al. [11] used marble slurry waste to produce sustainable materials in a circular economy. Torres et al. [12] studied the incorporation of granite cutting and polishing waste into building materials. Tolstoy et al. [13] studied in some detail the synergistic effects of different aggregates on the performance of green concrete. Klyuev et al. [14] developed high-strength fiber-reinforced concrete based on Russian aggregates. Bessmertny et al. [15] researched environmental thermal insulation composites using rock waste.

Of great importance is the cost of aggregates while taking care of the environment. An effective step along this path is the use of simultaneously produced rocks.

Granite, gravel, and limestone are the most popular and widespread sources of crushed stone, which are expensive, and their deposits are not available in all countries [10]. The search for alternative rock sources distinguishes four groups: ore-containing quartzites, quartz sandstones (QS), crystalline schists, and dyke rocks. In terms of reserves and physicomechanical properties, QS are of great interest [16]. In References [17,18,19,20], a detailed assessment was made of the quality of commonly available quartz-bearing rocks of sedimentary genesis: metamorphic, effusive igneous rocks, aluminosilicate rocks of the green shale degree of metamorphism, and carbonate rocks were studied. It has been established that with the identical mineral composition of rocks of the same name in petrographic groups, their energy potential can significantly differ [21,22]. The free internal energy contained in the structure of the raw material is determined by the imperfection of the crystal lattice of minerals, the inclusion of a mineral-forming medium, gas–air inclusions, the presence of an X-ray amorphous substance, surface morphology and texture, degree of crystallinity of minerals, dimension, etc. [23,24,25].

The goal of the paper was to study the possibility of using quartz sandstone, which is simultaneously mined rock, as aggregate for greener high-strength concrete. To achieve this goal, the following tasks were solved, the determination of the activity of natural radionuclides of the QS aggregate, the development of greener high-strength concrete using quartz sandstone (screening as fine aggregate and crushed stone as coarse aggregate), and the study of the physicomechanical properties and durability characteristics of developed concrete.

## 2. Raw Materials and Experimental Methods

### 2.1. Raw Materials Characterization

**Figure 1:**
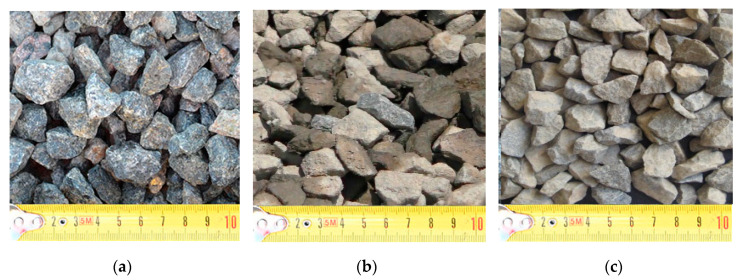
Coarse aggregates used: (**a**) granite, (**b**) gabbro diabase, (**c**) quartz sandstone (QS).

As a coarse aggregate, quartz sandstones of the Lebedinsky deposit (Russia) were used (Figure 1a), which are almost monomineralic rocks of a light gray and gray color. The rock-forming mineral is quartz. Usually, feldspar, muscovite, biotite, and fuchsite are found in small amounts. Quartz, cemented quartz-mica, and quartz pebbles are found in the base of the stratum in the form of lenses. The roof of QS has a sugar-like appearance with light and bright shades of color, and quartz marshalization is observed. Intergranular spaces and cracks are made of point iron hydroxides. All this speaks of the processing of these rocks by weathering processes. The thickness of the zone is 20 m. Therefore, the roof of quartz sandstone is not included in the calculation of reserves and aggregate from these rocks is not used for concrete production. However, an analysis of the physicomechanical tests results of QS from the Lebedinsky deposit indicates their suitability for use in the construction industry (Table 1). Granite and gabbro diabase from the Novopavlovsk deposit (Russia) were used as control materials for coarse aggregate (Figure 1b,c). To conduct tests of the physical and mechanical characteristics, the laboratory sample was dispersed into standard fractions: 5–10 mm (residue screening) and 10–20 mm (crushed stone) using laboratory sieves.

Thus, according to the main indicators, crushed stone from quartz sandstone meets regulatory requirements. The value of intergranular voidness of crushed stone from QS indicates the potential of improving its particle size distribution. Low water absorption of crushed stone from quartz sandstone reduces the water demand of concrete mixes.

**Figure 2:**
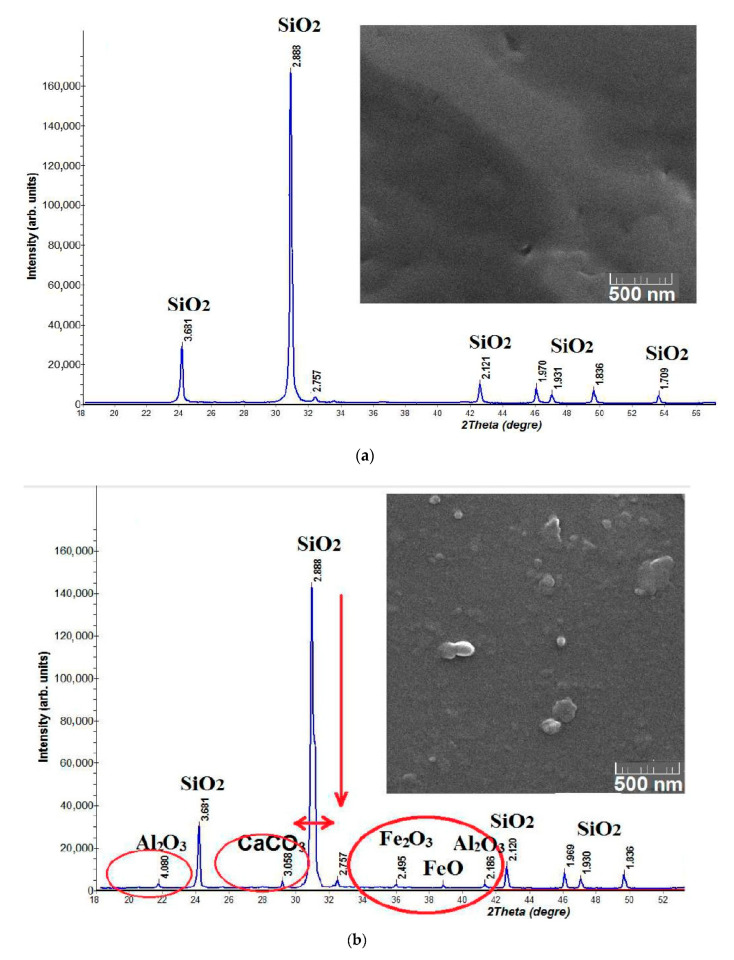
Features of the composition and structure: (**a**) quartz single crystal; (**b**) quartz sandstone of the green schist degree of metamorphism.

Samples of crushed stone from quartz sandstone consisted of 95% quartz of the green shale degree of metamorphism. Its quality assessment is based on the internal energy potential, which is determined by the imperfection of the crystal lattice of minerals, inclusions of a mineral-forming medium, gas–air inclusions, surface morphology, degree of crystallinity of minerals, dimension, etc. Figure 2 shows the differences between a quartz single crystal and quartz sandstone—mining waste, by the defectiveness of the quartz crystal lattice (XRD data), surface morphology (SEM images), and mineral composition (XRD data).

Thus, based on the study of the mineralogical composition, chemical composition, and structure of the QS mining waste of the Lebedinsky deposit, it can be concluded that all samples met the requirements for raw materials for the production of crushed stone. All experiments were carried out using Portland cement CEM I 42.5N (Belgorodsky Cement, Russia), satisfying the requirements of EN 197-1 [27,28,29]. The chemical composition of CEM I and aggregates used are listed in Table 2. Quartz sand with a fineness modulus of 1.9 was used as a fine aggregate. The Melflux 2651 F superplasticizer (SP) was used to thin the concrete mix.

### 2.2. Design of Concrete Mix

To identify the impact of the type of coarse aggregate on the technological properties of concrete mixes, concrete compositions were appointed from the condition of ensuring equal (in volume) consumption of crushed stone of all types. The slump of concrete mixes was 16 cm with a mass ratio of sand to the total aggregates equal to 0.4. To obtain the specified strength characteristics (concrete of classes C 25 and C 30), as well as to identify the potential capabilities of coarse aggregate from quartz sandstone, the cement consumption when selecting the composition of concrete varied from 350 to 400 kg/m^3^ (Table 3).

### 2.3. Experimental Methods

To evaluate the granulometric composition of the materials, a MicroSizer 201 laser particle analyzer (Scientific Instruments, Moscow, Russia) was used, which made it possible to determine particles with a size from 0.2 to 600 μm. The specific surface area of raw materials and binders was determined by gas permeability using a PSH-11 device. An ARL X’TRA device (Thermo Fisher Scientific, Waltham, MA, USA) was used for X-ray diffraction (XRD) analysis. The microstructure was studied using a high-resolution scanning electron microscope TESCAN MIRA 3 LMU (Brno, Czech Republic). The X-ray fluorescence (XRF) pattern was also investigated by scanning electron microscope.

The specific effective activity of natural radionuclides of crushed stone was researched using a Gamma Plus universal spectrometric complex (USC, Moscow, Russia) according to Russian State Standard GOST 30108-94 [28].

Determination of the compressive strength was carried out on specimen cubes with sizes 150 × 150 × 150 mm^3^ according to EN 12390-3:2009 [30]. The specimens hardened in the molds for 24 h, after which they were subjected to thermal–humid treatment according to the regime: 2 h—temperature rise to 65 °C; 10 h—isothermal exposure; 2 h—cooling to 20 °C.

The freeze–thaw resistance of the specimens was researched by the method of alternate freezing and thawing on cubes of 70 × 70 × 70 mm^3^ in accordance with EN 12390-9:2006 [31]. The specimens were immersed in water, first at 1/3 of the height for a day, then at 2/3 of the height for a day, and then completely immersed in water for two days. Then the specimens were placed in a Polair CV-105S freezer at a temperature of −18 °C. Each freezing cycle lasted 2.5 h, the thawing cycle at 20 °C lasted 2 h. The freeze–thaw resistance grade was evaluated by the value of the compressive strength after a certain number of freeze–thaw cycles. In this case, the decrease in mass should not exceed 2%, and the specimens should be free of cracks, chips, and flaking of the ribs.

**Figure 3:**
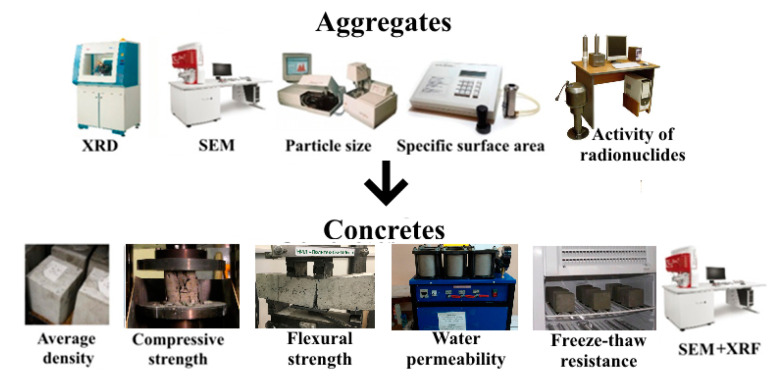
Flow chart of the studies.

The water resistance of concrete was estimated by the “wet spot” on the specimens—cylinders 100 × 100 × 150 mm^3^ in size. For this, a setup having 6 sockets was used. Water was supplied to the lower end part. Visual observation of the resistance to water with increasing pressure was conducted. Furthermore, a flow chart of the studies is provided in Figure 3.

## 3. Results and Discussion

### 3.1. Preparation of QS Aggregate

To maximize the disclosure of the stored energy of raw materials and their directed use while reducing the energy intensity of the technological process for producing building composites, the methods of chemical, thermal, and mechanical activation were used. The article used an energy-efficient, environmentally friendly method of non-thermal effects of electromagnetic pulses on the destruction mechanisms of quartz-containing raw materials. When current flows with extremely high power but moderately low energy through the matrix of mineral components, electrical breakdown channels as well as induced fracture zones are formed. This leads to the softening of mineral complexes with the formation of highly dispersed particles of increased activity and solves the problem of the destruction of raw materials. As a result, the number and nomenclature of hydration products increases and the density of the concrete increases; this leads to an increase in the mechanical properties of concrete.

According to the provisions of geomimetics science on the affinity of structures proposed by Lesovik [32], for designing an optimal composite, it is necessary that its components have the same linear expansion coefficients, deformation characteristics, adhesive characteristics, etc. Therefore, taking into account the genesis of the raw materials, it is possible to form a composite structure of a given quality [33]. Table 4 lists the deformative characteristics of quartz sandstone, allowing its use for aggregate in heavyweight concrete.

### 3.2. Ecological Safety of Quartz Sandstone

The environmental indicators of aggregate from quartz sandstone of simultaneously mined rocks from the Lebedinsky deposit were determined. The specific effective activity of natural radionuclides *A_eff_* was determined. In order to obtain the value of *A_eff_*, the specific activity of radium ^226^Ra—*A_Ra_*, thorium ^232^Th—*A_Th_*, and potassium ^40^K—*A_K_* was measured and summarized according to the formula: *A_eff_* = *A_Ra_* + 1.31*A_Th_* + 0.085*A_K_* (Table 5). It was found that the activity of natural radionuclides of the tested aggregate from quartz as was almost three times lower than that of the granite crushed stone of the Novopavlovsk deposit and four times lower than that of gabbro diabase. This indicates the indisputable advantage of the studied aggregate made of QS for use in cement systems.

### 3.3. Physicomechanical Properties of Concrete

In order to identify the influence of the type of coarse aggregate on the physicomechanical properties of concrete, the compositions were assigned according to Table 3 from the condition of ensuring equal consumption of crushed stone of three types. At the same time, the ratio of the mass of fine aggregate to the total mass of aggregates was taken to be 0.4 to ensure maximum packing density. Concretes of classes C 25 and C 30 were taken as targets to ensure the specified strength characteristics (Table 6).

The density of concrete mixes with quartz sandstone coarse aggregate was higher than in the case of granite and gabbro diabase. Thus, with constant consumption of materials, the replacement of the traditional coarse aggregate with quartz sandstone one reduces the weight of the structure, increasing its capacity for multi-story construction. At the same time, there was no increase in water demand and, as a result, a noticeable increase in the water–cement ratio. This eliminated the possibility of segregation of the concrete mix. In addition, there was an increase in compressive strength of 10–12% and flexural one by 15–25% compared with control specimens.

However, for specimens using quartz sandstone screening as a fine aggregate, there was a slight increase in water consumption. Obviously, this was due to the fact that the obtained quartz sandstone aggregates had more microcracks, which in turn increased the absorbent area for water. At the same time, in the next section, it is proved that due to the fact of these microcracks, a better adhesion strength of the cement paste to the aggregate—that is, a denser interfacial transition zone (ITZ)—is ensured.

### 3.4. Interfacial Transition Zone

**Figure 4:**
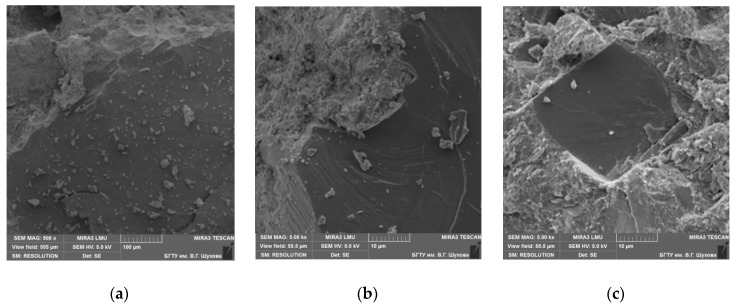
SEM images of the interfacial transition zone (ITZ) of quartz sandstone aggregate to cement paste: (**a**) general view; (**b**) microstructure; (**c**) borders on all sides.

**Figure 5:**
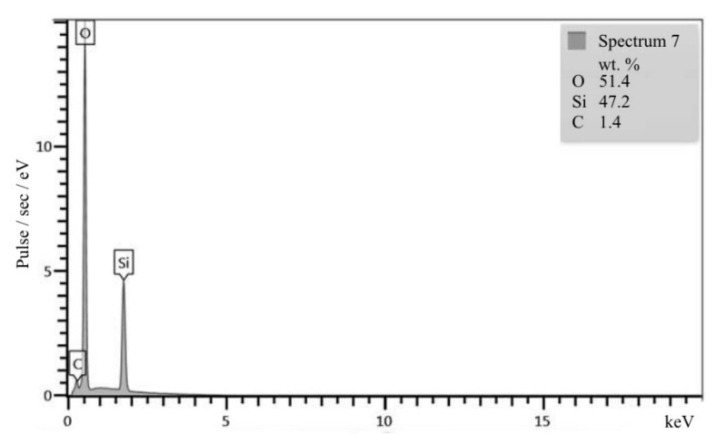
The XRF pattern of the ITZ of quartz sandstone aggregate to cement paste.

**Figure 6:**
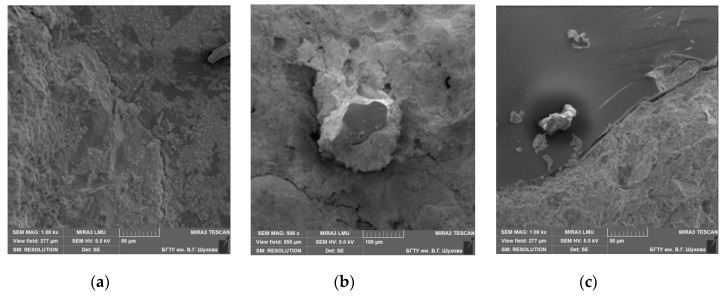
SEM images of the ITZ of granite aggregate to cement paste. (**a**) general view; (**b**) microstructure; (**c**) border.

**Figure 7:**
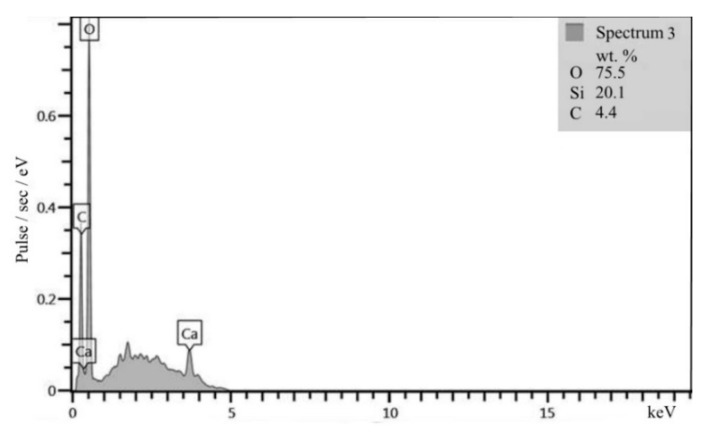
XRF pattern of the ITZ of granite aggregate to cement paste.

Granite consists of 30–35% quartz, approximately 60% feldspars, and the rest is mica. The cleavage of feldspars is perfect, but that of mica is very perfect. The adhesion to cement paste for feldspars and mica is very low, so granites cannot be used to produce high-strength concrete [17,18]. Gabbro diabase is an intrusive main rock consisting of plagioclase, augite, titanomagnetite, pyroxenes, and amphiboles [18,19]. These minerals also show low adhesion compared to quartz sandstone. The SEM images and XRF data explain the higher strength of concrete on quartz sandstone compared with the strength of concrete on granite crushed stone (Figure 4, Figure 5, Figure 6 and Figure 7). This is because quartz contained in QS has crystal lattice defects that are present in granite quartz in much smaller quantities. Therefore, the quartz of QS has a greater energy intensity and reactivity which determines its properties in comparison with granite. This is confirmed by testing prototypes of concrete on a crushed stone from quartz sandstone and granite. The structure of concrete with QS is characterized by the high density and strength of the interfacial transition zone (Figure 4a). By the finish of the hydration process, the pores are almost completely overgrown with crystals of hydrosilicate, hydroaluminate, and hydroferritic phases (Figure 4b), which perform a monolithic and reinforcing function, creating a strong network structure around the aggregate grains (Figure 4c). On the XRF pattern (Figure 5), the reflections of the new growths confirm the presence of calcium hydrosilicates and the absence of portlandite in the ITZ with crushed stone.

### 3.5. Weather Resistance

In the granite structure, quartz does not have as many crystal lattice defects as in quartz sandstone, which explains its lower reactivity. The ITZ is less dense; microcracks are present in it (Figure 6). This is confirmed by the nature of the new growths that are represented not only by calcium hydrosilicates but also by a significant amount of portlandite in the interfacial transition zone of crushed granite (Figure 7), which has a lower adhesion to the surface of the aggregate. Thus, a high degree of adhesion of the cement paste to the surface of the aggregate made of quartz sandstone was established in comparison with crushed granite.

For structural materials operating in environmental conditions, weather resistance is of great importance, which affects the durability characteristics. Table 7 lists the results of determining the freeze–thaw resistance and water resistance of the concrete. It was established that utilization of crushed stone from quartz sandstone provided greater freeze–thaw resistance and water resistance compared to the gabbro diabase or granite specimens. The test results indicated high durability of the obtained concrete. When cement consumption was 350 kg/m^3^, the water resistance of concrete corresponded to the W6 grade, and with an increase in cement consumption to 400 kg/m^3^, it increased to W12–W14. The freeze–thaw resistance of the concrete complied with grades F200 and F300.

Thus, concrete with a crushed stone from quartz sandstones exceeds a similar type of concrete from granite or gabbro diabase crushed stone in strength. The value of compressive strength corresponded to classes C25 and C30. An increase in compressive strength by 14% in concrete with quartz sandstone indicates the possibility of reducing the consumption of cement in such concrete.

It was established that the use of crushed stone from quartz sandstone provided greater freeze–thaw resistance and water resistance compared to the gabbro diabase or granite specimens. The test results indicated high durability of the obtained concrete. When cement consumption was 350 kg/m^3^, the water resistance of the concrete corresponded to the W6 grade, and with an increase in cement consumption to 400 kg/m^3^, it increased to W12–W14. The freeze–thaw resistance of the concrete complied with grades F200 and F300. Thus, concrete with crushed stone from quartz sandstones exceeded similar types of concrete with granite or gabbro diabase crushed stone in strength. The value of compressive strength corresponded to classes C25 and C30. An increase in compressive strength by 14% in concrete with quartz sandstone indicates the possibility of reducing the consumption of cement in such concrete.

### 3.6. Economic Efficiency

The economic efficiency of the results of the work was to reduce the cost of aggregates through the use of mine waste. In addition to the fact that this waste had no value, it can also generate income for the one who is utilizing it. The environmental objective achieved along with this makes it possible to consider the developed material with the use of aggregates from mine waste as cost-effective and environmentally effective.

## 4. Conclusions

Quartz sandstone is a mine waste; therefore, its use in construction allows both for reducing the cost of the concrete and contributing to the utilization of waste. The novelty of this study is the identification of models of the effect of QS aggregate on the physicomechanical, durability characteristics, and eco-safety of concrete. In the investigation campaign, the properties of greener high-strength concrete made with QS aggregate was investigated, bearing in mind the provisions of geomimetics science on the affinity of microstructures. Results were obtained on the properties of aggregate made of quartz sandstones activated by non-thermal effects of electromagnetic pulses as well as the heavyweight concrete based on it. On the basis of the results obtained, the following conclusions are listed:

− Quartz sandstone, which is a mine waste, can be used as aggregate for a high-strength, greenest concrete;
− The activity of natural radionuclides of the tested aggregate from quartz sandstone is 3–4 times lower than that of traditional types of crushed stone: granite and gabbro diabase;
− The use of a crushed stone from QS provides concrete with strength corresponding to grades C25 and C30. Mechanical properties of cement composite with coarse aggregate from quartz sandstone are 12–15% higher compared to concrete from traditional ones;
− The freeze–thaw and water resistance of concrete on crushed stone from QS provides a sufficient level of durability. The indicators of freeze–thaw and water resistance of concrete with a cement consumption of 300 kg/m^3^ corresponded to grades F200 and W8, respectively, and with a cement consumption of 400 kg/m^3^ corresponded to grades F300 and W14, respectively.

Furthermore, the perspectives for further scientific research can be directed to the possibility of obtaining aggregates from incidentally mined rocks for the creation of various building products. This will contribute to the expansion of the range of local raw materials used to obtain concrete components and the expansion of the field of research in the direction of studying the features of the processes of structure formation of composites using various fine and coarse aggregates.

## Figures and Tables

**Table 1:** The characteristics of coarse aggregate samples.

Characteristics of Crushed Stone	Test Sample			Standard Values by the Russian Standard GOST 26633-2015 [26]
	QS	Granite	Gabbro Diabase	
Fractions content, %				
10–20, mm	74.6	71.1	60.3	58–75
5–10, mm	25.0	27.8	39.5	40–25
<5, mm	0.4	1.1	0.2	2–0
The content of flaky and needle grains, %	33.3	8.5	17.5	<35
Bulk density, kg/m^3^	1413	1440	1532	-
True density, kg/m^3^	2635	2645	2762	2000–2800
Intergranular voidness, %	46.6	45.4	44.5	-
Water absorption, %	0.09	0.07	0.01	-
The content of clay and dust particles, %	0.8	0.9	0.4	<1
Grade of crushed stone by crushability	1200	1200	1400	300–1200

**Table 2:** The chemical composition of Portland cement and coarse aggregates used.

Chemical Composition	CaO	SiO_2_	Al_2_O_3_	Fe_2_O_3_	MgO	Alkalis
Cement, %	65.53	21.77	4.88	4.02	1.22	0.64
Quartz sandstone, %	0.56	94.32	2.63	0.42	0.68	0.97
Granite, %	2.52	74.32	14.52	2.41	0.73	6.25
Gabbro diabase, %	10.28	47.93	16.23	13.04	5.32	3.85

**Table 3:** Design of concrete mix.

Mix ID	Coarse Aggregate			Fine Aggregate		Cement	Water	SP
	Quartz Sandstone	Granite	Gabbro Diabase	Quartz Sand	Quartz Sandstone Residue Screenings			
Ref-350G	-	1210	-	590	-	350	145	4.5
Ref-400G	-	1210	-	590	-	400	166	4.5
Ref-350GD	-	-	1210	590	-	350	145	4.5
Ref-400GD	-	-	1210	590	-	400	166	4.5
350-1	1200	-	-	620	-	350	146	4.5
350-2	1200	-	-	-	620	350	154	4.5
400-1	1200	-	-	620	-	400	168	4.5
400-2	1200	-	-	-	620	400	172	4.5

**Table 4:** Deformative characteristics of quartz sandstone [7,8,9,10,11,12,13].

Characteristics	Unit	Values
Coefficient of thermal expansion, α_T_	1/°C	0.0000118
Abrasiveness, grade according to the Russian State Standard GOST 26633-2015 [27]	mg	IV, 18–30
Wear resistance, grade according to the Russian State Standard GOST 26633-2015 [27]	mm	III, 0.35–0.6
Impact strength, σ	MPa	306

**Table 5:** The specific effective activity of natural radionuclides of quartz sandstone in comparison with other aggregates.

Aggregate Type	Estimated Specific Effective Activity, Bq/kg
Quartz sandstone	36.4021
Granite	106.6387
Gabbro diabase	140.4507

**Table 6:** Physicomechanical properties of concretes.

Mix ID	Water/Cement Ratio	Average Density, kg/m^3^	Compressive Strength, MPa	Flexural Strength, MPa
Ref-350G	0.41	2370	31.0	3.2
Ref-400G	0.41	2392	45.0	3.5
Ref-350GD	0.41	2453	30.6	2.9
Ref-400GD	0.41	2509	43.2	3.2
350-1	0.41	2310	35.4	3.6
350-2	0.44	2350	34.1	3.8
400-1	0.42	2360	46.3	3.7
400-2	0.43	2370	46.8	3.9

**Table 7:** Freeze–thaw resistance and water resistance of the concrete.

Mix ID	Compressive Strength (28 Day), MPa	Water Resistance Grade	Compressive Strength, MPa, After Freezing and Thawing Cycles				Freeze–Thaw Grade
			150	200	250	300	
Ref-350G	31.0	W6	30.2	29.5	-	-	F200
Ref-400G	45.0	W12	-	-	43.7	42.8	F300
Ref-350GD	30.6	W6	30.0	29.1	-	-	F200
Ref-400GD	43.2	W12	-	-	41.9	40.4	F300
350-1	35.4	W8	34.8	33.9	-	-	F200
350-2	34.1	W8	33.8	32.7	-	-	F200
400-1	46.3	W14	-	-	44.0	43.2	F300
400-2	46.8	W14	-	-	44.3	43.8	F300
